# Integrating inflammatory serum biomarkers into a risk calculator for prostate cancer detection

**DOI:** 10.1038/s41598-021-81965-3

**Published:** 2021-01-28

**Authors:** Amirhossein Jalali, Michael Kitching, Kenneth Martin, Ciaran Richardson, Thomas Brendan Murphy, Stephen Peter FitzGerald, Ronald William Watson, Antoinette Sabrina Perry

**Affiliations:** 1 UCD Conway Institute of Biomedical and Biomolecular Science, Dublin, Ireland; 2 UCD School of Medicine, University College Dublin, Dublin, Ireland; 3 School of Mathematical Sciences, University College Cork, Cork, Ireland; 4 UCD School of Biology and Environmental Science, University College Dublin, Dublin, Ireland; 5 Randox Teoranta, Co, Donegal, Ireland; 6 School of Mathematics and Statistics, University College Dublin, Dublin, Ireland; 7 Randox Laboratories Ltd, 55 Diamond Road, Crumlin, Co., Antrim, BT29 4QY Ireland

**Keywords:** Diagnostic markers, Prostate cancer, Risk factors, Prostate

## Abstract

Improved prostate cancer detection methods would avoid over-diagnosis of clinically indolent disease informing appropriate treatment decisions. The aims of this study were to investigate the role of a panel of Inflammation biomarkers to inform the need for a biopsy to diagnose prostate cancer. Peripheral blood serum obtained from 436 men undergoing transrectal ultrasound guided biopsy were assessed for a panel of 18 inflammatory serum biomarkers in addition to Total and Free Prostate Specific Antigen (PSA). This panel was integrated into a previously developed Irish clinical risk calculator (IPRC) for the detection of prostate cancer and high-grade prostate cancer (Gleason Score ≥ 7). Using logistic regression and multinomial regression methods, two models (Logst-RC and Multi-RC) were developed considering linear and nonlinear effects of the panel in conjunction with clinical and demographic parameters for determination of the two endpoints. Both models significantly improved the predictive ability of the clinical model for detection of prostate cancer (from 0.656 to 0.731 for Logst-RC and 0.713 for Multi-RC) and high-grade prostate cancer (from 0.716 to 0.785 for Logst-RC and 0.767 for Multi-RC) and demonstrated higher clinical net benefit. This improved discriminatory power and clinical utility may allow for individualised risk stratification improving clinical decision making.

## Introduction

Prostate cancer (PCa) is the most common non-cutaneous cancer in men in the Western world^1^. A prostate tissue biopsy is a key step in the diagnosis of PCa. However the decision to refer a patient for a biopsy is challenging, as TRUS biopsies are associated with significant morbidity^2^. Clinicians usually base this decision on serum prostate specific antigen (PSA), abnormal digital rectal exam (DRE)^3^ and increasingly multiparametric magnetic resonance imaging (mpMRI) as well as other factors, such as family history and previous biopsy results. PSA lacks specificity^1^ and has led to over-diagnosis and over-treatment of clinically indolent disease and a large number of unnecessary biopsies in men^4^ and the mpMRI PROMIS trial did show that there is still a chance of missing clinically significant disease at PI-RADS scores of 1 or 2^5^.

Improved detection methods for high-grade significant disease that would reduce unnecessary biopsies are highly sought. Risk stratification of men suspected of PCa and high-grade significant disease would allow clinicians and patients to make a more informed decision on whether or not to biopsy. Risk calculators that utilise patient clinical data have already been developed for cardiovascular disease^6^ and stroke^7^. There are several guidelines which suggest a risk adapted approach that considers clinical information along with serum PSA should be used to predict PCa risk^8^. Previous risk calculators have been developed and assessed such as the European Randomised Study of Screening for Prostate Cancer Risk Calculator (ERSPC-RC)^9^ and the Prostate Cancer Prevention Trial Risk Calculator (PCPT-RC)^10,11^ in large multi-institutional cohorts. However, the accuracy of the risk score nomograms to detect high grade cancer (Gleason score ≥ 7) are ~ 69–79% for both PCPT and the ERSPC.

The use of multiple serum biomarkers may be the key to improving the identification of significant disease. Commercial tests that utilise serum biomarkers are already on the market, such as the Prostate Health Index (PHI)^12^, which comprises total PSA, free PSA and [-2] proPSA^13^ and the 4 K score, which assesses total PSA, free PSA, intact PSA, and human kallikrein-related peptidase 2^14^. Although promising, these commercial tests are currently not widely routinely used, as there is still uncertainty as to their utility and interpretation in a clinical setting. Previous studies have shown that the inclusion of serum and Urine biomarkers improves on risk calculators. Our own studies have shown that the addition of the PHI score to an Irish clinical risk calculator improved the accuracy of the Irish risk calculator^15^. The urinary biomarkers prostate cancer antigen 3 and the gene fusion product of transmembrane protease serine 2 with the transcription factor v-ets erythroblastosis virus E26 oncogene homolog (TMPRSS2-ERG) also improved the accuracy of ERSPC-RC^16^. It is clear that the use of multiple biomarkers improves on the accuracy of risk calculators.

It is well recognised that inflammation plays a causal role in the development of several types of cancer^17^ and there is direct evidence of an inflammatory microenvironment^18^ and higher inflammatory marker levels effecting a greater PCa risk^19^. This environment is associated with impaired differentiation of prostate epithelial cells^20^ and aberrant basal to luminal differentiation promoting cancer initiation^21^.

The aim of this study was to investigate the utility of inflammatory serum biomarkers combined with clinical information for the detection of (i) PCa and (ii) high-grade PCa in patients that are suspected of having PCa.

## Materials and methods

### Patient cohort and sample collection

The study cohort consisted of 436 Caucasian Irish men referred for a TRUS biopsy on the basis of an elevated PSA and/or abnormal DRE between April 2012 and June 2016. Blood samples (9 mL) were collected in a serum separator tube prior to biopsy and processed within 3 h of collection. Samples were centrifuged at 1500×*g* for 15 min at room temperature. Serum (~ 3 mL) was removed and stored at − 80 °C until further analysis. Patients were classified as either biopsy-negative (having no detectable PCa) or biopsy-positive (having detectable PCa) and further sub-divided into low-grade (Gleason score 6) and high-grade (Gleason score 7 or above)^15^ disease. The clinicopathologic details of the cohort are summarised in Table 1.

**Table 1:** Clinical Features of the Patient Cohort.

	All Patients	Biopsy positive	Biopsy negative	P Value	High-grade PCa	Low-grade PCa	P Value
Patients, n (%)	436	211 (48.4)	225 (51.6)		146 (69.2)	65 (30.8)	
**Age in years**							
Median	63.9	65.2	63.2	0.096^†^	65.6	62.2	0.045^†^
Mean	63.3	63.8	62.8	0.156*	64.4	62.2	0.051*
Range	(41.70–84.96)	(41.70–84.96)	(41.77–79.13)		(41.7–85.0)	(45.6–77.0)	
**DRE, n (%)**							
Normal	263 (60.3)	110 (52.1)	153 (68.0)	0.002^¥^	70 (48.0)	40 (61.5)	0.184^¥^
Not recorded	9 (2.1)	7 (3.3)	2 (0.9)		5 (3.4)	2 (3.1)	
Abnormal	164 (37.6)	94 (44.6)	70 (31.1)		71 (48.6)	23 (35.4)	
**PSA in ng mL**^**−1**^							
Median	6.5	7.0	6.0	< 0.001^†^	8.2	5.9	< 0.001†
Mean	14.0	21.9	6.6		28.4	7.0	
Range	(0–1,400)	(0–1,400)	(0.5–31.40)	0.027*	(0–1,400)	(0.54–29.3)	0.032*
**Family history of PCa, n (%)**							
Positive	75 (17.2)	4 (20.9)	31 (13.8)	0.067^¥^	33 (22.6)	11 (16.9)	0.451^¥^
Negative	361 (82.8)	167 (79.1)	194 (86.2)		113 (77.4)	54 (83.1)	
**Previous negative biopsy, n (%)**							
Yes	87 (20.0)	29 (13.7)	58 (25.8)	0.002^¥^	17 (11.6)	12 (18.5)	0.266^¥^
No	349 (80.0)	182 (86.3)	167 (74.4)		129 (88.4)	53 (81.5)	
**Gleason score, n (%)**							
6	–	63 (29.9)	–	–	–	63 (100)	–
7		99 (46.9)	–		99 (67.8)	–	
8		26 (12.3)	–		26 (17.8)	–	
9		20 (9.5)	–		20 (13.7)	–	
10		3 (1.4)	–		3 (2.1)	–	

*Student’s T-test.

^¥^ Pearson’s chi squared test.

^†^Wilcoxon Rank test.

Sample collection and processing were ethically approved by the St James Hospital and Mater Misericordiae University Hospital ethics committees. The patient information leaflet and consent form were written and constructed in line with best practice and the EU Data protection Directive and Data protection Acts 1988 and 2018 and approved by the two ethics committees. All patients gave written informed consent agreeing to participate in the study. All steps were carried out in accordance with national guidelines and regulations.

### Biomarker analysis

In total, analysis of 20 biomarkers was performed. The Evidence Investigator platform (Randox) uses sandwich chemiluminescent immunoassay methods for the simultaneous detection of multiple analytes. Two multiplexed Evidence Investigator biomarker panels were employed; the Cytokine and Growth Factors High Sensitivity Array (Cat. No. EV 3623) for IL-1α, IL-1β, IL-2, IL-4, IL-6, IL-8, IL-10, VEGF, IFN-γ, TNF-α, MCP-1 and EGF^22^ and the Adhesion Molecules Array (Cat. No. EV3519) for VCAM-1, ICAM-1, E-Selectin, P-Selectin, L-Selectin^23^. Biochip analyses were performed according to the manufacturer’s instructions. Briefly, serum samples diluted where appropriate, reconstituted calibrators and assay specific quality controls utilised in duplicate were incubated on the biochip. Following washing, detector conjugate solution was applied to the biochip and binding was revealed using chemiluminescent detection. Concentration of all analytes in the samples were calculated using a nine-point calibration curve by the Evidence Investigator analyser. Individual assay runs were deemed to have passed if the measured values for the quality control samples were within the specified range for each of the target values for each analyte, as per kit instructions. Values measured below the lower limit of detection were taken as zero.

IL-18 was measured in all serum samples by ELISA (Cat. No. ILE10068, Randox) according to the manufacturer’s instructions. Serum samples, calibrators and quality control samples were added to each well in duplicate. The IL-18 standard provided in the kit was reconstituted in deionised water to make up the calibrators and the two quality control samples (312.5 pg mL^−1^ and 56.25 pg mL^−1^). Total PSA and Free PSA were determined in all samples using the Roche COBAS 8000 system according to manufacturer’s instructions at Randox Clinical Laboratory Services (Antrim, UK).

Even though PSA values were available for each patient we included its analysis as the patients were recruited from different clinics and the pre-biopsy PSA values attained using various platforms. Therefore, serum PSA of the patients were reanalysed on the single platform.

Clinical information: Age, Family history, DRE and prior negative biopsy were collected as part of the study from the patients chart or at time of recruitment.

### Statistical analysis

#### Basic analysis of patient information

Basic statistical analysis of the study population’s characteristics was performed using GraphPad Prism (ver. 5.0). Descriptive statistics were performed in the dataset, which was divided into those with and without a PCa diagnosis and high-grade PCa (> = Gleason 7) versus all other patients. The unpaired Student’s t-test and the Wilcoxon Rank test were used to investigate the significant difference in means and medians of continuous variables, respectively. Pearson’s chi-squared test was also performed to studying the significant difference for categorical variables.

#### Risk calculator model development and performance

Development of the risk calculator for the prediction of PCa and high-grade PCa were performed in R software version 3.4.3^24^. Logistic and multinomial regression methods were used to model the linear and nonlinear effects of serum biomarkers combined with clinical information (age, DRE, family history of PCa, previous negative biopsy). These two modelling strategies are considered as relevant approaches to stratify patients to high-grade PCa, low-grade PCa or those without PCa. The stepwise method was applied as the variable selection technique to integrate potentially relevant biomarkers into the risk calculator. In both methods, the probabilities for each patient were modelled through the log odds of risk factors which were then transformed into probabilities and assigned a percentage risk for each patient. Internal validation is built into the cross-validation approach to prevent overfitting of the data by using tenfold cross validation.

The final models for diagnosis of PCa and/or high-grade PCa were compared to the Irish prostate risk calculator (IPRC) which has been previously developed and outperformed the available risk calculators in the Irish population^25^. Accuracy of the models was determined using the area under the curve (AUC) calculated from the Receiver Operator Curve (ROC) by plotting the sensitivity and specificity at each of its risk thresholds. Comparison of ROC curves took place via the method described by DeLong et al.^26^. Decision-curve analysis was undertaken to examine the potential net benefit of the application of each model over the benefit offered by the strategies of performing a biopsy in all patients and performing a biopsy in none^27^. Calibration plots were plotted to represent the agreement between the observed incidence of cancer visually and predicted risk^28^. The Chi-Square Hosmer–Lemeshow test was used to assess the goodness of fit of models, where a p < 0.05 indicates a poor agreement between the predicted risk and observed incidence of cancer and a poorly calibrated model.

## Results

### Baseline cohort characteristics

This was a retrospective biomarker study intended to improve the detection of clinically relevant disease. The clinical endpoint of the study was the histopathological findings from the TRUS biopsy. The study cohort consisted of 436 patient biopsies, of which 211 (48%) were diagnosed with PCa with different Gleason scores (Table 1).

In Table 1, the univariate effects of 'DRE' and 'Previous negative biopsy' were statistically significant in detecting PCa, and 'age' for detecting high-grade PCa. The effect of 'PSA' was also significant in both cases. This implies that if patients are older, have higher PSA, abnormal DRE or did not have a previous negative biopsy, (on average) they have more chance of PCa and high-grade PCa.

### Statistical modelling

Descriptive analysis of all biomarkers assessed is presented in Table 2.

**Table 2:** Descriptive analysis of serum biomarkers (median and interquartile range) grouped by biopsy and grading outcomes.

Median (IQR)	All patients (n = 436)	Biopsy positive (n = 211)	Biopsy negative (n = 225)	P Value	High-grade PCa (n = 146)	Low-grade PCa (n = 65)	P Value
tPSA (ng mL^−1^)	6.28 (4.87)	7.15 (5.72)	5.51 (4.13)	< 0.001	7.92 (9.11)	5.69 (3.10)	< 0.001
fPSA (ng mL^−1^)	1.02 (0.95)	1.03 (1.20)	1.01 (0.82)	0.112	1.10 (1.69)	0.85 (0.65)	0.017
IL-1α (pg mL^−1^)	0.09 (0.14)	0.09 (0.14)	0.09 (0.16)	0.655	0.09 (0.14)	0.09 (0.14)	0.988
IL-1β (pg mL^−1^)	0.44 (0.75)	0.49 (0.80)	0.16 (0.73)	0.389	0.44 (0.75)	0.56 (0.82)	0.253
IL-2 (pg mL^−1^)	0.0 (1.15)	0.0 (1.08)	0.0 (1.21)	0.645	0.0 (1.14)	0.0 (0.98)	0.916
IL-4 (pg mL^−1^)	1.33 (0.57)	1.34 (0.56)	1.28 (0.58)	0.730	1.36 (0.62)	1.28 (0.45)	0.074
IL-6 (pg mL^-1^)	1.12 (1.05)	1.16 (1.26)	1.04 (0.88)	0.069	1.34 (1.51)	0.95 (0.51)	0.005
IL-8 (pg mL^−1^)	7.24 (5.17)	7.38 (5.07)	7.05 (5.18)	0.395	7.60 (4.78)	6.86 (5.23)	0.176
IL-10 (pg mL^−1^)	0.54 (0.34)	0.57 (0.33)	0.51 (0.35)	0.145	0.56 (0.32)	0.59 (0.40)	0.911
IL-18 (pg mL^−1^)	185 (165)	193 (148)	179 (174)	0.216	205 (152)	179 (105)	0.105
VEGF (pg mL^−1^)	72.1 (78.9)	76.3 (78.6)	68.2 (80.0)	0.193	78.8 (80.2)	75.6 (69.8)	0.630
IFN-γ (pg mL^−1^)	0.18 (0.30)	0.18 (0.31)	0.18 (0.28)	0.242	0.20 (0.22)	0.16 (0.29)	0.208
TNF-α (pg mL^−1^)	1.92 (0.90)	1.94 (0.84)	1.86 (0.95)	0.391	1.98 (0.88)	1.82 (0.77)	0.391
MCP-1 (pg mL^−1^)	155 (96.1)	154 (97.9)	155 (94.9)	0.751	150 (92.2)	166 (117)	0.350
EGF (pg mL^−1^)	5.34 (12.8)	5.82 (12.4)	4.64 (13.4)	0.794	5.51 (12.9)	6.48 (10.2)	0.630
VCAM-1 (ng mL^−1^)	645 (211)	666 (212)	635 (199)	0.121	692 (208)	612 (190)	0.016
ICAM-1 (ng mL^−1^)	272 (80.6)	275 (76.2)	270 (78.9)	0.283	283 (79.2)	265 (66.7)	0.010
E-Selectin (ng mL^−1^)	23.3 (10.7)	25.2 (12.9)	22.7 (9.47)	0.016	26.4 (12.5)	22.3 (10.9)	0.027
P-Selectin (ng mL^−1^)	164 (60.8)	197 (50.5)	192 (72.8)	0.301	198 (51.0)	193 (46)	0.993
L-Selectin (ng mL^−1^)	896 (230)	898 (214)	894 (251)	0.977	899 (210)	890 (207)	0.486

The p-value of Wilcoxon Rank test indicates whether the observed differences in median for each biomarker is significant.

Integrating serum biomarkers with the clinical risk factors using multinomial and logistic models identified 8 biomarkers (TNFα, VEGF, IL1α, IL1β, ICAM-1, E-selectin, P-selectin, L-selectin) with 4 (IL1α, IL1β, E-selectin, P-selectin) biomarkers identified in both models, to confer significant additional predictive ability. Two separate risk calculators were developed to predict PCa and high-grade PCa using a multinomial model (Multi-RC) and a logistic model (Logst-RC) where Table 3 presents the models and Table 4 evaluates the model performances. We also built models using the biomarkers alone for both the multinomial model (Multi-bio) and a logistic model (Logst-bio) presented in Table 4 which showed no significant improvement over the clinical model (IPRC).

**Table 3:** Summary of Multi-RC and Logst-RC models using odds ratio, standard error and p-value for each risk factor in the model.

	Multi-RC (A)			Multi-RC (B)			Logst-RC (C)			Logst-RC (D)		
	Odds ratio	Std. Error	p-value	Odds ratio	Std. Error	p-value	Odds ratio	Std. Error	p-value	Odds ratio	Std. Error	p-value
Age	0.997	0.023	0.884	1.031	0.020	0.137	1.021	0.017	0.238	1.038	0.020	0.058
DRE (Abnormal)	1.203	0.324	0.569	1.545	0.276	0.115	1.498	0.240	0.093	1.501	0.265	0.125
DRE (Missing)	4.084	1.051	0.181	2.790	1.016	0.313	3.250	0.949	0.214	1.659	0.905	0.576
Family history (Positive)	1.339	0.408	0.475	1.341	0.342	0.392	1.251	0.296	0.450	1.303	0.322	0.412
Previous biopsy (Yes)	0.605	0.385	0.192	0.318	0.344	< 0.001	0.426	0.284	0.003	0.366	0.333	0.003
TNFα	0.213	0.891	0.083	1.022	0.075	0.770	–	–	–	–	–	–
TNFα^¥^	22.06	1.848	0.094	1.108	0.178	0.564	–	–	–	–	–	–
VEGF	–	–	–	–	–	–	–	–	–	1.003	0.002	0.104
VEGF^¥^	–	–	–	–	–	–	1.214	0.108	0.073	–	–	–
IL1α	1.605	0.282	0.093	1.578	0.258	0.077	1.527	0.253	0.095	–	–	–
IL1α^¥^	0.921	0.072	0.257	0.856	0.065	0.017	0.894	0.055	0.041	–	–	–
IL1β^¥^	1.115	0.049	0.028	1.057	0.042	0.193	1.068	0.036	0.06	–	–	–
ICAM-1	0.954	0.004	< 0.001	0.991	0.002	< 0.001	–	–	–	–	–	–
ICAM-1^¥^	89,574.6	0.409	< 0.001	11.806	0.341	< 0.001	–	–	–	–	–	–
E-selectin	1.006	0.020	0.768	1.052	0.016	0.001	1.029	0.013	0.029	1.052	0.014	< 0.001
P-selectin	1.004	0.003	0.232	0.996	0.003	0.129	0.983	0.009	0.084	0.995	0.003	0.057
P-selectin^¥^	–	–	–	–	–		22.22	1.963	0.114	–	–	–
L-selectin	–	–	–	–	–	–	0.993	0.003	0.037	0.993	0.003	0.028
L-selectin^¥^	–	–	–	–	–	–	404.6	2.961	0.043	1088.2	3.213	0.030
FPSA	0.813	0.510	0.685	1.634	0.345	0.155	–	–	–	1.709	0.303	0.077
FPSA^¥^	1.056	0.394	0.891	0.487	0.319	0.024	0.285	0.277	< 0.001	0.103	0.474	< 0.001
TPSA	–	–	–	–	–	–	1.092	0.041	0.034	–	–	–
TPSA^¥^	1.357	0.238	0.200	2.704	0.238	< 0.001	3.400	0.384	0.001	11.43	0.342	< 0.001
FTPSA^¥^	0.778	0.307	0.414	0.180	0.252	< 0.001	–	–	–	–	–	–

^¥^ The non-linear effect of the predictor using a log transformation.

**Table 4:** The discriminative ability of IPRC, Multi-bio, Logst-bio, Multi-RC and Logst-RC using the areas under the curve (AUC) and 95% confidence interval of the calculated probabilities.

Risk calculators	AUC	95% CI	P-value (compared to IPRC)
**IPRC**			
PCa	0.656	(0.609–0.711)	–
High-grade PCa	0.716	(0.663–0.769)	–
**Multi-bio**			
PCa	0.686	(0.637–0.735)	0.392
High-grade PCa	0.749	(0.701–0.798)	0.271
**Logst-bio**			
PCa	0.708	(0.659–0.756)	0.127
High-grade PCa	0.770	(0.724–0.816)	0.068
**Multi-RC**			
PCa	0.713	(0.664–0.761)	0.038
High-grade PCa	0.767	(0.719–0.815)	0.036
**Logst-RC**			
PCa	0.731	(0.684–0.778)	0.006
High-grade PCa	0.785	(0.738–0.831)	0.003

The p-values indicate if each model significantly improve the IPRC.

The odd ratios of the Multi-RC model for detecting low-grade (column A) and high-grade PCa (column B) compared to not detecting PCa are presented in Table 3. We combined odd ratios of low grade and high grade PCa to evaluate the performance of the Multi-PC model for detecting PCa and High-grade PCa and these are presented in Table 4 and show a significant improvement above the IPRC model. The model variables consist of Age, DRE, Family History, previous biopsy, PSA, TNF-a, IL-1a, IL-1b, ICAM-1, E-Selectin, P-Selection, Free PSA (FPSA) and Free to total PSA (FTPSA).

The odd ratios of the Logst-RC model for detecting PCa compared to not detecting PCa (column C) and detecting high-grade PCa compared to low-grade or not detecting PCa (column D) are presented in Table 3. The model performance for detecting PCa and High grade PCa are presented in Table 4 and showed a significant improvement above the IPRC model. The model variables consist of Age, DRE, Family History, previous Biopsy, PSA, VEGF, IL-1a, IL-1b, E-Selectin, P-Selectin, L-Selectin and FPSA.

To give some insight into the clinical significance of the study, we selected thresholds manually based on the Youden index criteria. Using the threshold of 0.3 for high-grade Logst-RC (and the threshold of 0.275 for high-grade Multi-RC) with would have resulted in saving 71.2% (72.6%) of the biopsies at the cost of delaying the diagnosis of 27.9% (33.8%) of the high-grade cancers. The negative predictive value of the test results below this threshold would be 0.833 (0.828).

### Model performance

**Figure 1:**
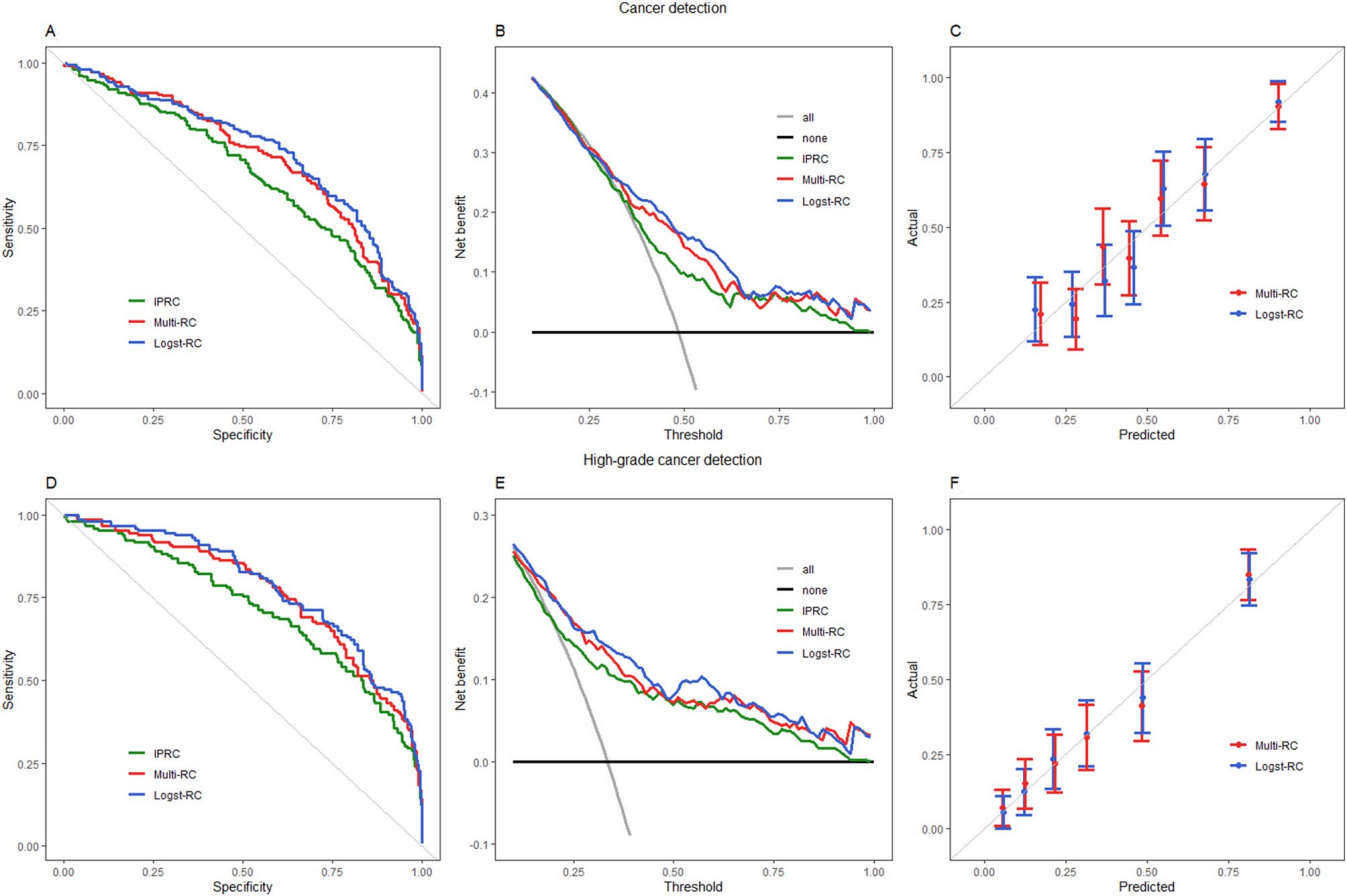
The receiver operating characteristic (ROC) curves (**A**,**D**) and decision curves (**B**,**E**) represent the discriminative ability of IPRC (green), Multi-RC (red) and Logst-RC (blue) in diagnosis of cancer (**A**,**B**) and high-grade cancer (**D**,**E**). Calibration curves are represented in (**C**,**F**).

Table 4 represents the discriminative abilities of both risk calculators for the diagnosis of PCa and high-grade PCa using AUC. Multi-RC showed an AUC of 0.7126 and 0.7671 and Logst-RC an AUC of 0.7308 and 0.7847 for diagnosis of PCa and high-grade PCa respectively. This significantly improved the predictive ability of the IPRC model, as demonstrated in the ROC in Fig. 1A,D.

Figure 1 shows the decision curve analyses of the clinical utility of both models in detecting PCa (Fig. 1B) or high-grade PCa (Fig. 1E). For detecting PCa there was an improved net benefit for the threshold ranges of 0.35 to 1.0 and for detecting high-grade PCa there was an improved net benefit for the threshold ranges of 0.15 to 1.0 compared to the IPRC—clinical model alone. The calibration curves (Fig. 1C,F) show good agreements between predicted probabilities and the actual outcome indicating that all models are well calibrated, which have been confirmed by the (non-significant) Hosmer–Lemeshow results.

Integrating the panel of serum biomarkers with the clinical risk factors outperform the previously developed Irish risk calculator^25^. Logst-RC has shown slightly higher improvement when internally validated; however, further validation in an independent cohort will be required in order to confirm improvements and identify the most appropriate model and could be employed to select the best clinically accepted threshold to be used in practice.

## Discussion

In this study, we have utilised a retrospective approach to show that the integration of inflammatory serum biomarkers into the clinical risk factors significantly improves the discriminatory power and clinical utility of the clinical risk factors alone for PCa and high-grade PCa (Gleason Score ≥ 7). This suggests that the Multi-RC or Logst-RC models would improve the detection rate and/or reduce unnecessary biopsies compared to the IPRC risk calculator based on clinical features alone. These models demonstrated consistently higher net benefits over different preferences of wanting or avoiding a biopsy^29^ and following further validation and threshold selection could have clinical utility.

Chronic inflammation is associated with the development of many cancers including PCa and is possibly playing a role in its formation and development^30^. In the current study we identified a number of inflammatory mediators that increased the prediction of PCa and high-grade PCa when integrated with the current clinical features compared to clinical features alone. These included TNF-α, VEGF, IL-1α, IL-1β, ICAM-1, E-Selectin, P-Selectin and L-selectin. There is evidence in the literature that some of these mediators are associated with tumour development and progression including PCa. VEGF has been shown to be overexpressed in patients with colorectal^31^ and PCa. Fryczkowski et al. demonstrated that VEGF concentrations were significantly higher in the PCa groups compared to the BPH patient group however on multiple logistic regression analysis VEGF was not an independent predictor of PCa and did not add to the clinical features alone^32^. Soluble adhesion molecules ICAM-1 and the selectins have been shown to be increased in Breast^33^ and colorectal cancer^31^ but there is no evidence in PCa to date. TNF-α levels have also been correlated with disease stage in breast cancer^34^ but there is no evidence that TNF-α serum levels are associated with high grade of lethal PCa at the time of diagnosis of localised disease as well as IL-1α and IL-1β^35^. The power of our study is that we evaluated a number of inflammatory serum mediators and built a model selecting the biomarkers that gave the best prediction of PCa and high-grade PCa.

The multinomial regression modelling approach identified a single combination of biomarkers for the risk assessment of PCa and high-grade PCa. However, two different sets were selected to estimate the risk of PCa and high-grade PCa in the logistic regression approach. The use of logistic regression helps to access the partial effect of the biomarkers on either detecting PCa or high-grade PCa, while the use of multinomial regression reduces the standard errors^36^. Both of these methods are employed in previous studies, including, the European risk calculator (ERSPC-RC^9^) used the logistic regression approach, and two American risk calculators (PCPT-RC^10^ and PBCG-RC^11^) are developed using the multinomial regression.

The use of a logarithm transformation for some biomarkers in the model (e.g. IL-1β) represents a nonlinear effect of the biomarker on the risk, which indicates that a small change in the biomarker is critical. In contrast, the linear effect of some biomarkers in the model (e.g. E-selectin) represents that a change in any value of the biomarker has the same importance. The use of both linear and logarithm effects of the biomarkers in the model (e.g. IL-1α) indicates that, although any change in the biomarker is important, a small change in the values of the biomarkers are more critical.

A limitation of the study is not having PSA density as a variable which is part of the ERSPC-PC^8^. We did not have access to the prostate volume data for this study at the time of patient recruitment as the Irish health care setting did not facilitate the collection of prostate volume until the TRUS biopsy was carried out.

## Conclusion

Our study has demonstrated that as both models are well calibrated and utilise variables that are available from the patient (Age, Family history, DRE and Previous Biopsy) and assessed from their blood sample they are appropriate for individualized risk assessment. Both models show a statistically significant improvement above the IPRC justifying the addition of the serum biomarkers and their clinical use. Selecting the best model requires additional validation cohorts which would be used to independently validate and identify the best model and select the appropriate thresholds which are clinically accepted and maximize their discrimination and clinical benefit.

## Data Availability

Data is available to other researchers on written request to the corresponding author.
